# Morphological features of basivertebral foramen among different age groups: Recognition in spine

**DOI:** 10.3389/fsurg.2023.1115654

**Published:** 2023-03-21

**Authors:** Qiang Wang, Benyu Jin, Jianfeng Zhang

**Affiliations:** Sir Run Run Shaw Hospital, School of Medicine, Graduate School, Zhejiang University, Hangzhou, China

**Keywords:** morphological, basivertebral foramen, vertebral body, measurement, spine

## Abstract

**Background:**

Basivertebral foramen (BF) is a vessel and nerve passage in the posterior wall of vertebral body (VB). Our goal was to report BF's morphological characteristics in different age groups of mainland Chinese adults and to evaluate the relationship between BF's morphology and factors such as age, gender, heavy work, size and level of VB.

**Methods:**

We enrolled 300 adults among persons who came to our hospital for health examination. We measured BFs and VBs’ parameters on T1 weighted sagittal lumbar spine MR images. We also assessed following potential predictors: gender, body height, body weight, body mass index, alcohol use, habits of smoking and drinking, type of work (physical work or non-physical work). A stepwise multivariate linear regression analysis was conducted to identify predictors of BF's height.

**Results:**

People above 60 have significantly bigger BFHr than those in young adulthood and in the middle ages at all five levels, while they have shallowest BFs, especially at L3. Multiple linear regression resulted in a formula that accounted for 30.1% of the variability in the height of basivertebral foramen. Significant predictors included: gender, age, level, vertebral height and heavy work.

**Conclusion:**

Age is the highest weight in all factors on the height of BF. BF is closer to the upper endplate. The BF was relatively higher and deeper in the female lumbar spine. Heavywork results in lower BF. Last but not the least, as we supposed, BF gets shallower and higher compare to VB with age.

**Level of evidence:**

Prognostic level III. See instructions for authors for a complete description of levels of evidence.

## Introduction

**Figure 1:**
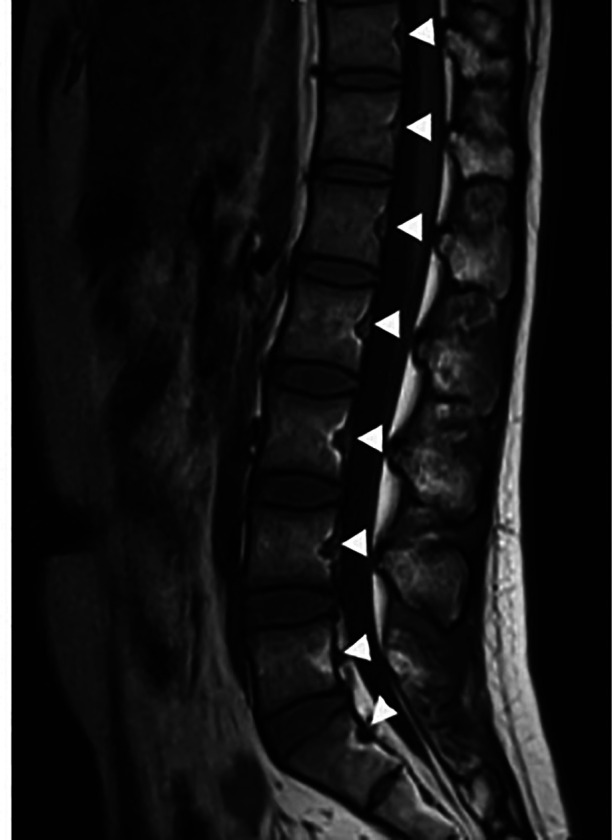
BFs in the vertebrae from T11 to S1 (arrow heads).

Basivertebral foramen (BF) is a bone defect in the posterior wall of vertebral body (VB), between the pedicles, containing basivertebral veins, arteries and nerves (Figure 1). BF existed in every vertebra of the spine (1–5). It has a bigger size than other vessel passage of bone (6). The intravertebral circulation links to vertebral venous system *via* BF, through so-called basivertebral vein, single or sometimes with two separate tributaries. All the venous system is valveless, and it eventually drains to Vena Cava (7–9). Studies implied that BFs had a relationship with lots of diseases and phenomenons, such as tumor metastasis, postero-superior fragment in burst fracture of thoracolumbar spine and cement leakage of percutaneous vertebroplasty or kyphoplasty (type B cement leakage) (10). Our former research also revealed the presence of a connection between the basivertebral foramen and the intravertebral cleft (11), and the possibility of direct cement leakage into the spinal canal through the basivertebral foramen when PKP is performed in senior patients of osteoporotic vertebral fracture with intravertebral cleft (12).

To this day there's no systemically study about the morphological features of BF, although which is the fundament of the recognition of the relationship between BF and vertebral diseases. Spinal metastasis was thought to be happen firstly in pedicle arch, because x-ray examination showed the malignant colony firstly settling in this region (13). But Algra et al. (14) found that, explicated by CT, spinal metastasis often happened firstly at posterior part of VB because of BV, since basivertebral vein served as an entrance site for hematogenous spread of metastases (9, 15–18). Algra et al. (14) also described that the lumbar region of the spine was the area most frequently affected by metastases; next were the thoracic and cervical portions. Lumbar spine bears the largest loading in the whole spine, while a big bony defect in the VB such as BF would impose important biomechanical influence on the VB, especially in the lumbar spine. Thus, the BFs of the lumbar spine became the first subjects we studied among the spine.

Spine degenerates as human being getting aged. Bone density decreases, and the shape of vertebrae also changes with age. In series of reports by Erichsen between 1976 and 1978, lumbar vertebrae showed relative lowering and broadening of their bodies with age (19–21). As to easterners, Kim et al. (22) found that VB height decreased with age in thoracic vertebrae (T5, T6) and lumbar vertebrae (L4, L5) among Koreans. As the VBs get less bone mass and flattened with age, we suppose that BFs would get shallower and higher compare to VBs. To prevent the influence of size change of BVs among different age groups, we calculated relative values of BFs to BVs and compared these relative values.

## Materials and methods

### Patient population

Between January 2012 and November 2015, many adults came to our hospital for regular health examination including a Magnetic Resonance Imaging scan of lumbar spine. Exclusion criteria were spine diseases including infection and tumor, deformity, severe osteoporosis, hypertension and vessel diseases. Among these healthy adults, 300 were randomly included in this study and then divided into three age groups (under 30, 30–60, and over 60), and in each group were 50 females and 50 males.

### Measurements and deﬁnitions

**Figure 2:**
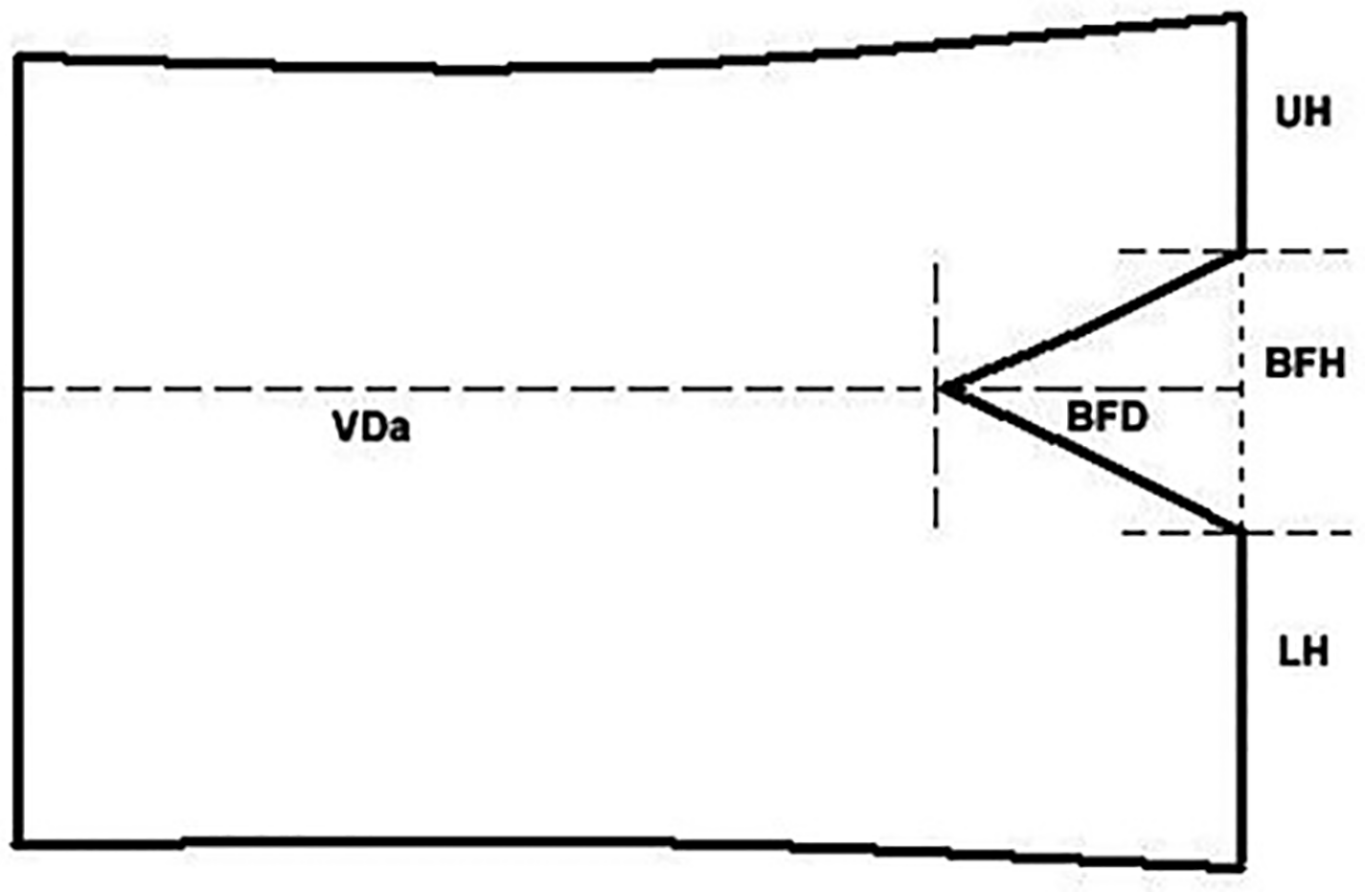
BFs’ parameters in a drawing of VB. The BFH is the distance between the two crossover points of BF and posterior wall of VB. The BFD is the distance of anterior point of BF to the line crossing the two crossover points. Along the line, the distance of the upper crossover point to the upper endplate called UH, and the distance of the lower crossover point to the lower endplate called LH. The distance of anterior point of BF to the anterior margin of VB is VDa.

We measured BFs’ parameters on T1 weighted sagittal images. We measured BF's height (BFH) and depth (BFD), vertebral body height (VH) and depth (VD) of every lumbar vertebral body (Figure 2). The BFH is the distance between the two crossover points of BF and posterior wall of VB. The BFD is the distance of anterior point of BF to the line crossing the two crossover points. Along the line, the distance of the upper crossover point to the upper endplate called UH, and the distance of the lower crossover point to the lower endplate called LH. The distance of anterior point of BF to the anterior margin of VB is VDa. So the VH equals to the sum of UH, LH and BFH (VH = UH + LH + BFH), and the VD equals to the sum of VDa and BFD (VD = VDa + BFD). In addition, the ratio of BFH to VH called BFHr, and the ratio of BFD to VD called BFDr (Figure 2).

Two clinicians carried out the work independently and measured every VB for three times. The averages of every clinician's three values were collected for statistic analysis. Before measuring, the clinicians discussed and agreed on the measurement method. In case of the margins of a few VBs and BFs were hard to find because of the reasons such as image quality or soft tissue interference, two clinicians reach a consensus about the margins after discuss.

### Predictors evaluated

We also assess following potential predictors: gender, body height, body weight, body mass index, alcohol use, smoking, type of work (physical work or non-physical work).

### Statistical analysis

BFH, UH, LH, BFD, VDa, BFHr, UHr, LHr and BFDr were compared in three age groups using KW test or ANOVA analysis, and in two gender groups using Mann-Whitney test or *t*-test, and in five vertebral level groups using KW test. Wilcoxon test or paired *t*-test was used to compare UH and LH. A stepwise multivariate linear regression analysis was conducted to identify predictors of basivertebral height.

Statistical significance was defined as a probability level of ≤0.05. All analyses were performed with SPSS 19.0 statistical package (SPSS, Inc., Chicago, IL).

### Source of funding

This work was supported by grants from the National Natural Science Foundation of China.

## Results

**Figure 3:**
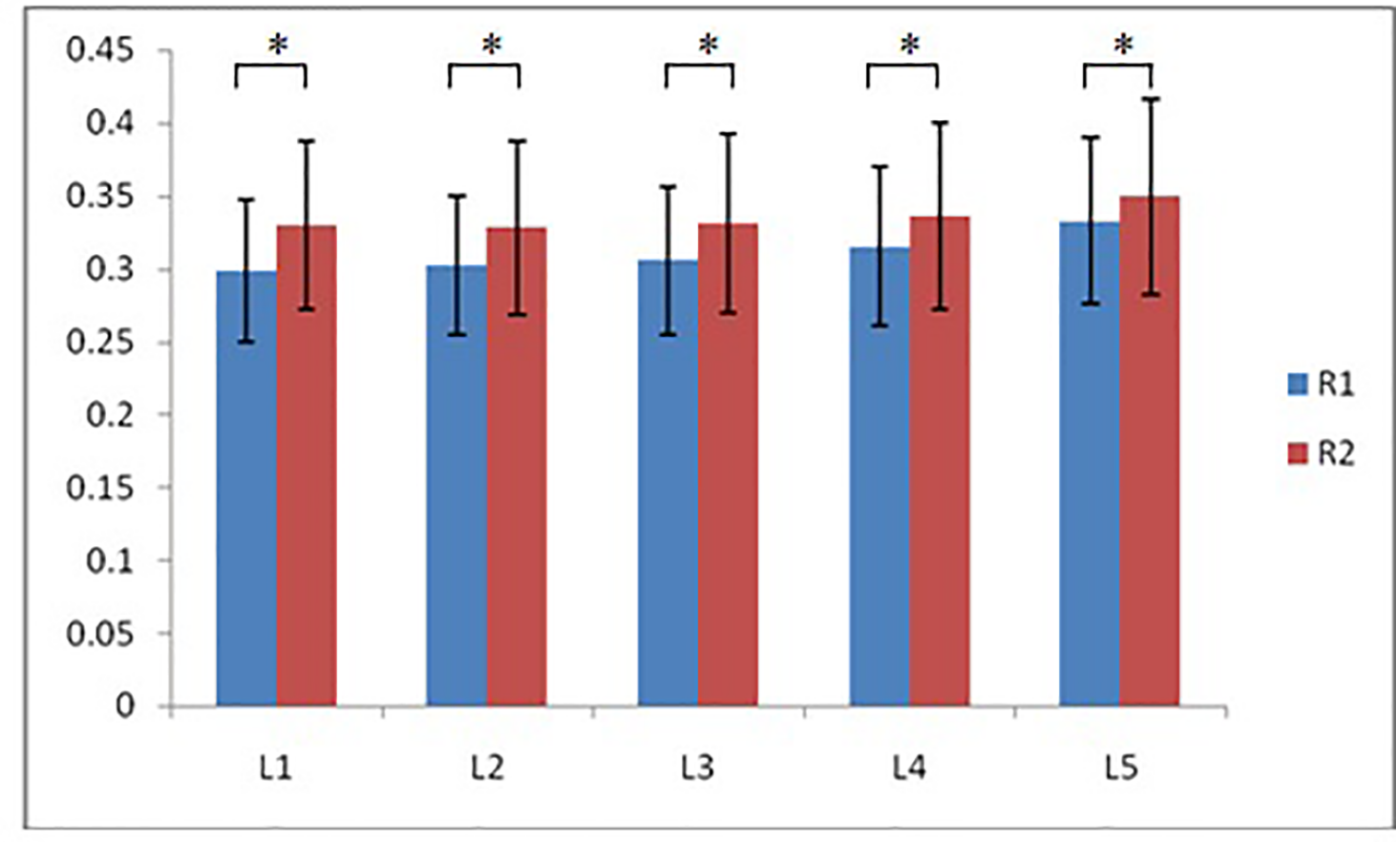
Compared with the lower endplates, the basivertebral foramen are more close to the upper endplates at all five measured levels (R1: the ratio of distance between BF and upper endplate to VB height; R2: the ratio of distance between BF and lower endplate to VB height).

**Figure 4:**
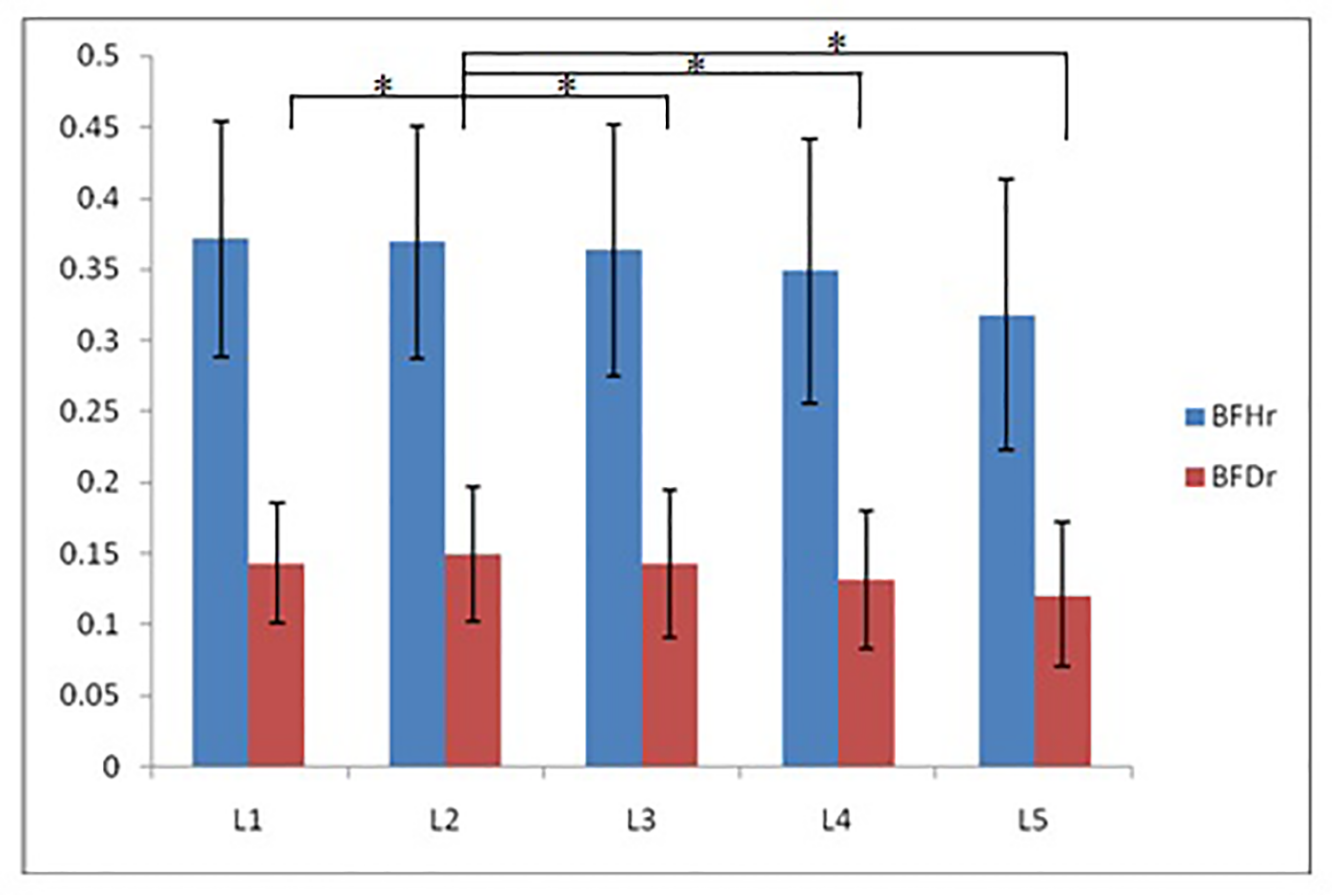
The BFHr at the lower level tends to be relatively smaller, while BFDr at L2 is the biggest among five vertebral levels.

**Figure 5:**
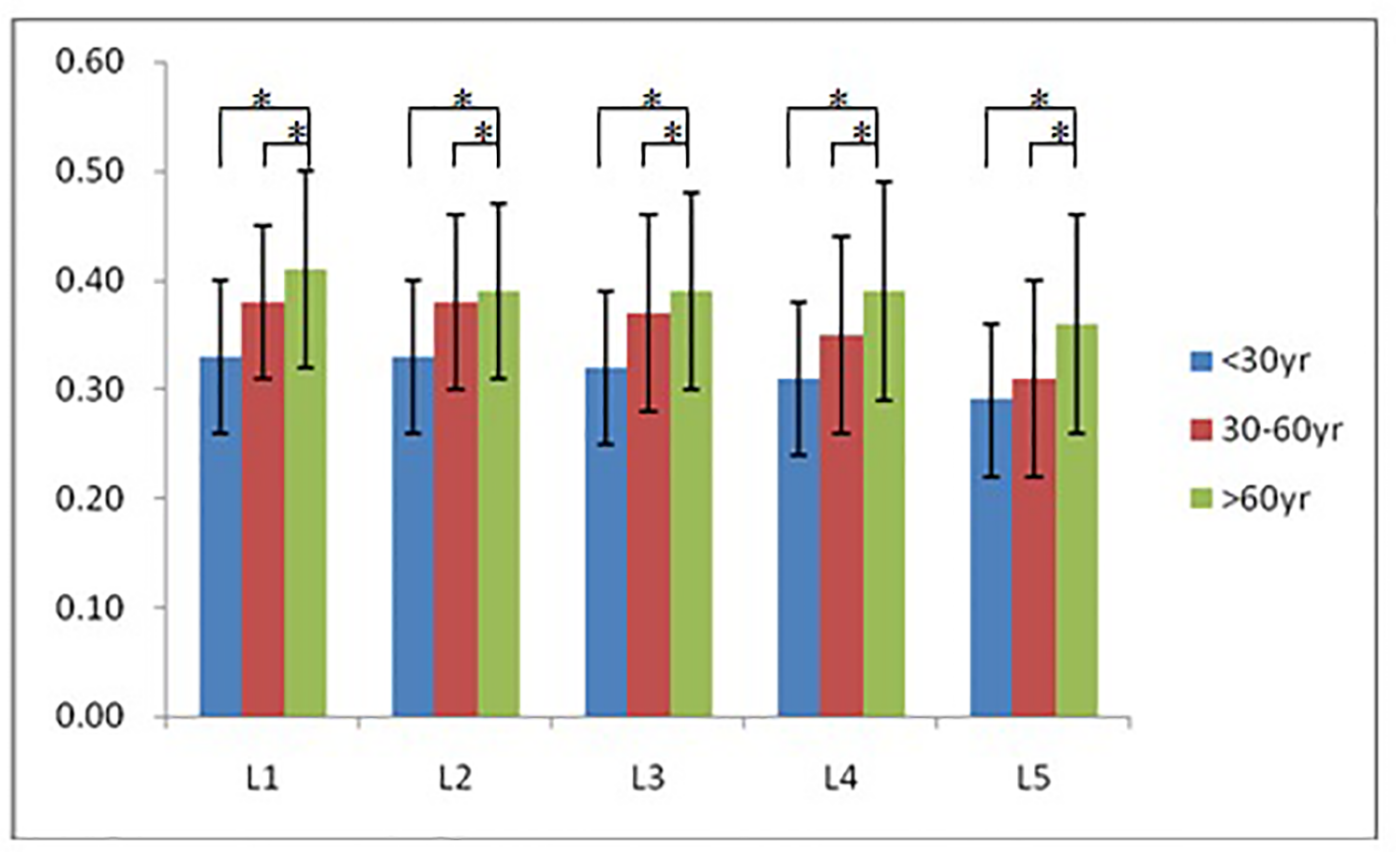
People above 60 have significantly bigger BFHr than those in young adulthood and in the middle ages at all five levels.

**Figure 6:**
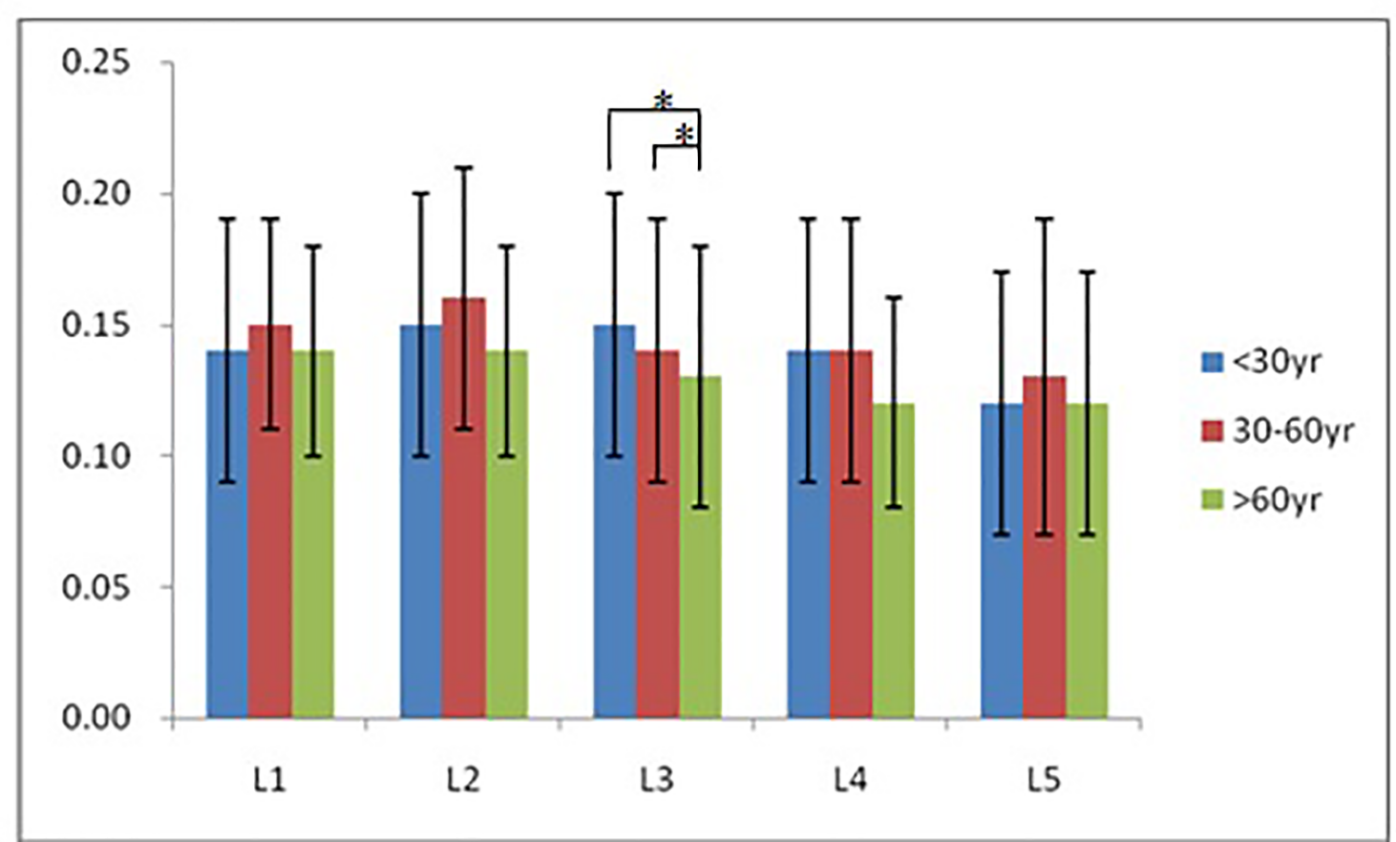
People above 60 have significantly smallest BFDr only at L3, but tend to have shallowest BFs among three groups.

**Figure 7:**
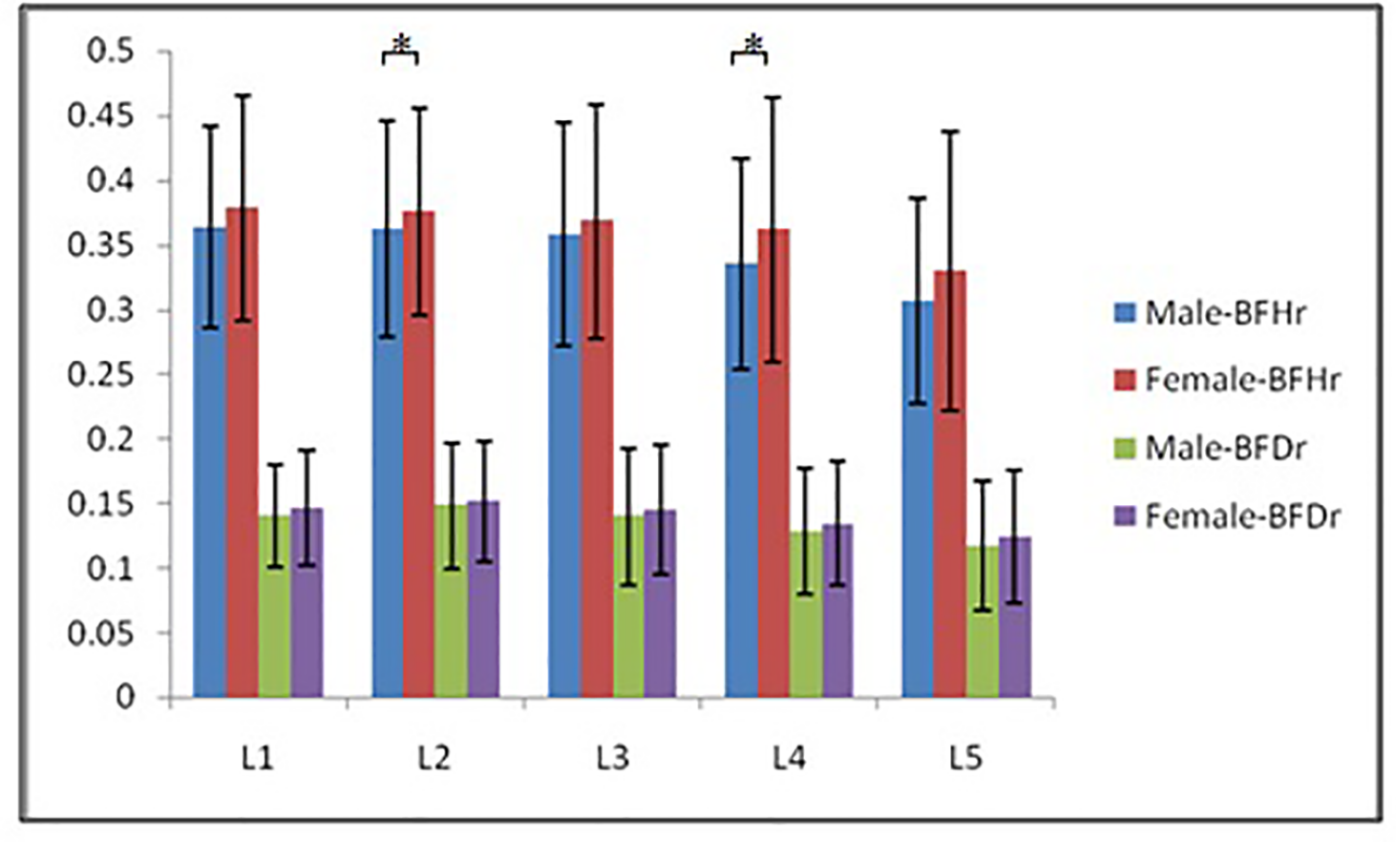
Females have higher BFHr and deeper BFDr than males in lumbar vertebrae, which only BFHr at L2 and L4 show statistically significant difference.

According to morphological characteristics of basivertebral foramen, we classified them as 3 types: triangular type (65%), trapezoid type (24%) and irregular type (11%). The bone interval exsist in 6% of the basivetebral foramen. The average basivertebral height is 9.2 ± 2.5 mm, which is approximately 1/3 of vertebral height at the same level, whilst the average basivertebral depth is 4.4 ± 1.6 mm. Compared with the lower endplates, the basivertebral foramen are statistically more close to the upper endplates at all five measured levels (*P* < 0.05, Figure 3). The BFHr at the lower level tends to be relatively smaller, while BFDr at L2 is the biggest among five vertebral levels (*P* < 0.05, Figure 4). People above 60 have significantly bigger BFHr than those in young adulthood and in the middle ages at all five levels, while they have shallowest BFs, especially at L3 (*P* < 0.05, Figures 5, 6). Females have higher BFHr and deeper BFDr than males in lumbar vertebrae, which only BFHr at L2 and L4 show statistically significant difference (*P* < 0.05, Figure 7). Multiple linear regression resulted in a formula that accounted for 30.1% of the variability in the height of basivertebral foramen. As detailed in Table 1, significant predictors included: gender, age, level, vertebral height and heavy work.

**Table 1:** List of significant predictors.

	Unstandardized coefficients		Standardized coefficients	*t*	Sig.
	B	Std. error	Beta		
Constant	−.161	.034		−4.700	.000
Gender*	.254	.054	.237	4.691	.000
Age	.360	.034	.371	10.659	.000
Vertebral level**	−.228	.035	−.237	−6.491	.000
Vertebral height	.263	.041	.262	6.453	.000
Heavy work***	−.068	.032	−.068	−2.089	.037

* 1 = male, 2 = female.

** 1 = L1, 2 = L2, 3 = L3, 4 = L4, 5 = L5.

*** 1 = with heavy work, 2 = without heavy work.

## Discussion

Although many studies have investigated the anatomy, circulation, innervation and biomechanical features of spine, the BV is still a blind zone of research. Our study and some previous studies showed that BV, such a big bony defect, lies in middle column according Denis and Ferguson's (23–25) three column theory of spine, anterior to spinal canal and posterior to the center of VB, a little closer to upper endplate. The size and location of BV could tell the truth of many clinical phenomenons such as burst fracture and type B cement leakage in PVP and PKP. The strain around the BV is the highest within the entire VB and along the surface of VB (26, 27), so posterior regions of the VB had greater bone volume, more connections, reduced trabecular separation and more plate-like isotropic structures than anterior regions (28). Although the superior part of posterior BV had better bone quality, the factors of the thinner upper endplate and closer BV seemed to share more in burst fracture, resulted in posterior superior fracture fragment (28–32). As the ratio of BF's height to VB's height largens according to aging, the posterior region and wall of VB would burden more strain, as a result, which would become fragile under violence. Thus, we can easily found that osteoporotic compression fracture of aging VB resulted in flattened VB while the younger non-osteoporotic VB were wedge-shaped after compression fracture. Thoracolumbar spine (T11–L1) (33, 34) is the most often fractured in spine, results from which previous studies argued that this span of spine is a transitional section and with lots of motion. In our study, the BFHr of L1 was the largest among the lumbar vertebrae, which could be a cause of the high risk of fracture of L1.

Cement leakage happened quite often in PKP/PVP (35–37), especially under CT examination post-operatively (38–40). Extravertebral cement leakage *via* the basivertebral veins (so-called type B) and fractured cortex (so-called type C) are more than other type. Type B is more dangerous than type C (37), and would be harmful (7, 39, 41). Because of BF, unilateral injection of cement had more cement leakage than bilateral injection (42). The needle head would get closer to BF when surgeon tried to put it into the center of VB. Our study showed that the depth of BF is up to 15% of anteroposterior diameter of VB, so the region anterior to the posterior 15% of VB is safe for cement injection. This is consistent with the founding of Yeom et al.'s study (37), who declared that cement existed in posterior 1/5 of VB might be a leakage.

The basivertebral nerves which might be the sole passage that enabled intravertebral innervations made BF more important (43, 44). Intravertebral nerves clustered at the center of VB, after descended from basivertebral nerves. That's why a little cement injected into the VB could also ameliorate symptom in osteoporotic-vertebral-fractured patients by PVP/PKP, just like larger volume (45). As mentioned before, the calibration of BF is larger than other nutritional foramen of bone. The existence of basivertebral nerves could make a reason. Although VB's volume changed under compressive loading *via* flow through the BF, and the basivertebral vein deformed in the course (46), it's still unimaginable that it needs a passage of over 1/3 of VB's height (except for L5 which is 29%). However, sufficient space for basivertebral nerves is essential not only to tolerate the enlarged BF but also to take precautions against deformation of BF that resulted from loading, fracture, hyperostegeny and so on.

The vascular system of VB is extremely complicated (10, 47). The central vascularity of the vertebral body which originated from vessels entering BF (48, 49) is established during fetal development, while peripheral vessels persist after adolescence (50). Tortuosity of the central intravertebral arteries and presence of vessels around the vertebral periphery increases with age (50) and is thought to be variable based on degeneration and nutrient availability. In our study, the depth of BF tended to be less as aging, especially in L3, which had significant tendency. It could be implied that as peripheral vessels increasing when aging, the nutrition supply *via* central vessels attenuated, so the depth of BF decreased. It is also possible that shallowed BF, because of microfractures or creep deformation, compromised blood supply of VB, causing VB degeneration under malnutrition, and then resulting in peripheral vessels infiltration. However, central vessels are still one of the main nutritional passages for the BFs in the aged VBs were just a little shallower than those in younger ones. Algra et al. reported that disappearing BF might have relation with bone marrow disease of VB (51).

Female had higher and deeper BF than male, although only L2 and L4's rates of BF's height to VB's height showed significance. Although female have smaller body size and VB size, the BFs were relatively bigger than male. Heavy work was a negative factor of BF height, which is intuitively understood, attributed to increased creep deformation and microfractures of VB.

This study has the following merits: the factors analyzed in our study are easily and reliably determined in the clinical practice. Besides factors that could be obtained by inquiry, the vertebral height could be acquired from cheap and time-saving x-ray examination, with acceptable radiation.

**Figure 8:**
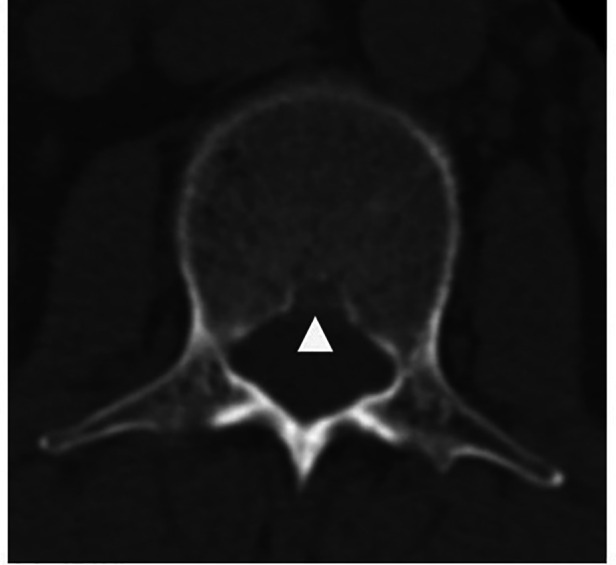
A wide BF is shown in CT scan (arrow head).

However, some limitations could not be ignored. First, as a bony defect, BF is most explicit in CT. In our region, the cost of MRI is just a little more expensive than CT, but with sparse radiation. Although MRI is time-consuming, patients still prefer MRI examination about spine. It costs us a lot of time to calibrate the parameters while carefully identified the low signal of cortex of VB, but it is not as accurate as calibration in CT image. Second, we couldn't get the width of BF. The images only contained transverse section of intervertebral discs. Although occasionally clinical CT examination of spine showed large width of BF (Figure 8), it needs further prospective study to reveal the truth. Third, some factors that might affect the BF such as blood pressure and race were not included in the study. Whether and how blood pressure, especially hypertension, influences BF is an interesting topic, we would carry out such a study to explicit the relationship.

## Conclusion

Age is the highest weight in all factors on the height of BF. BF is closer to the upper endplate. The BF was relatively higher and deeper in the female lumbar spine. Heavywork results in lower BF. Last but not the least, as we supposed, BF gets shallower and higher compare to VB with age.

## Data Availability

The original contributions presented in the study are included in the article/Supplementary Material, further inquiries can be directed to the corresponding author.
